# O10 - Rupatadine is effective and safe in the treatment of Chronic Spontaneous Urticaria (CSU) in pediatric patients (2-11 years old)

**DOI:** 10.1186/2045-7022-4-S1-O10

**Published:** 2014-03-14

**Authors:** Paul Potter, Essack Mitha, Laszlo Barkai, Alejandro Domenech, Eva Santamaria, Josep Giralt, Iñaki Izquierdo, Marcus Maurer

**Affiliations:** 1 UCT Lung Institute, Cape Town, South Africa; 2 Newtown Clinical Research, Johannesburg, South Africa; 3 BAZ Megyei Korhaz, Miskolc, Hungary; 4 Clinical Research Uriach, Barcelona, Spain; 5 Dermatology and Allergy, Charite Hospital, Berlin, Germany

## Background

Rupatadine is an anti-H1/PAF antagonist that has proven to be effective and safe in adults/adolescents for Allergic Rhinitis (AR) and Urticaria. In addition, Rupatadine has been recently licensed for the symptomatic treatment of AR in children aged 6-11 years. The aim of this study was to assess the efficacy and safety of Rupatadine oral solution in children with Chronic Spontaneous Urticaria (CSU).

## Methods

A randomized, double blind, multicenter, parallel group, placebo and active controlled clinical trial was conducted in children aged 2-11 year suffering from CSU. Patients were randomly allocated to one of the treatment groups: Rupatadine 1mg/mL, Desloratadine 0.5mg/ml, or placebo oral solutions. Dosing was done according to patients’ weight (2.5 ml > 10 Kg - 25 Kg or 5 ml > 25 Kg). Patients received the study medication during 6 weeks (42 days) and symptoms were scored daily in the patient diary. There was a safety follow up period of 6 weeks (42 days) after the end of treatment. The primary efficacy variable was the change in the 7-day cumulative children adapted Urticaria Activity Score (UAS7) over the treatment period. Other efficacy assessments included the mean number of wheals (MNW) score, mean pruritus (MPS) score and Children Dermatology Life Quality Index (CDLQI). Adverse events were recorded through the study (84 days).

## Results

A total of 199 patients were included in the ITT analysis: 63 in the Rupatadine group, 69 in the Desloratadine group, and 67 in the placebo group. The UAS7 was found to be significantly reduced in the Rupatadine group (-55.8%; p=0 .001) and the Desloratadine group (-48.4%; p= 0.013) as compared to placebo (-30.3%). Also, CDLQI scores were significantly improved in both active treatment groups as compared to placebo. The overall incidence of adverse events was similar among treatments groups.

## Conclusion

Rupatadine, in accordance with the current CSU guideline recommendations, is an effective and safe choice in CSU in 2-11 yr children.

